# “They are no longer really flashbacks; they are memories”: a qualitative study of women’s experiences of a brief intervention targeting childbirth-related intrusive memories

**DOI:** 10.3389/fpsyg.2026.1891666

**Published:** 2026-07-06

**Authors:** Déborah Fort, Camille Deforges, Joan Lalor, Ulrike Rimmele, Elisabeth Schobinger, Antje Horsch

**Affiliations:** 1 Faculty of Biology and Medicine, Institute of Higher Education and Research in Healthcare, University of Lausanne, Lausanne, Switzerland; 2 School of Nursing and Midwifery, Trinity College Dublin, Dublin, Ireland; 3 Emotion and Memory Laboratory, Faculty of Education Sciences and Psychology, University of Geneva, Geneva, Switzerland; 4 Swiss Centre for Affective Sciences (CISA), University of Geneva, Geneva, Switzerland; 5 Institute of Psychology, University of Lausanne, Lausanne, Switzerland; 6 HESAV School of Health Sciences - Vaud, HES-SO University of Applied Sciences and Arts Western Switzerland, Lausanne, Switzerland; 7 Neonatology Service, Department Woman-Mother-Child, Lausanne University Hospital, Lausanne, Switzerland

**Keywords:** behavioural intervention, childbirth, intrusions, intrusive memories, maternal care, posttraumatic-stress disorder

## Abstract

**Introduction:**

Childbirth-related posttraumatic stress symptoms (CB-PTSS), including intrusive memories (CB-IM), affect a substantial proportion of mothers and can have lasting negative consequences for themselves and their families. A single-session behavioural intervention combining a childbirth memory recall in the maternity ward and a visuospatial task involving the videogame Tetris, has shown preliminary efficacy in reducing CB-IM and CB-PTSS. However, how women subjectively experience this intervention, whether they perceive it as potentially beneficial, and what mechanisms they believe may contribute to change remain unknown. This qualitative study, embedded within a multicentre single-blind waitlist-controlled randomised trial conducted in Switzerland, aimed to explore: (1) how women experienced the two components of this intervention (i.e., recall and Tetris); (2) what changes they perceived following the intervention; and (3) what mechanisms they attributed to these changes.

**Methods:**

Seventeen participants were recruited from a multicentre, single-blind, waitlist-controlled randomised trial in which they receive a single-session behavioural intervention targeting CB-IM. A maximum variation sampling strategy based on self-reported changes in CB-IM frequency following the intervention was utilised. Semi-structured interviews were realised and analysed using inductive content analysis.

**Results:**

Recalling the childbirth memory at the maternity ward was experienced as emotionally intense (i.e., stressful, destabilising), yet simultaneously meaningful for most participants. Playing Tetris elicited initial surprise or scepticism but was then experienced as engaging and soothing by most participants. Participants reported a broad range of perceived changes, most notably a reduction in CB-IM number, alongside qualitative changes in CB-IM, and greater acceptance of the childbirth memory. Perceived mechanisms of change were predominantly talking or writing about the childbirth experience.

**Conclusion:**

These findings support the broad clinical benefits of this single-session intervention for women reporting established CB-IM. This study also highlights the importance of non-specific therapeutic factors may play a meaningful role in this intervention’s benefits.

## 1 Introduction

Feeling supported, in control, safe, and respected are central components of a positive childbirth experience (Leinweber et al., 2023). When these conditions are not met, women may experience intense distress linked to interactions or events that can contribute to a traumatic childbirth experience (Leinweber et al., 2022). As with other traumatic events, a traumatic childbirth can lead to the development of posttraumatic stress symptoms (PTSS), affecting approximately 12% of mothers (Heyne et al., 2022). PTSS include intrusive memories (IM), avoidance, hyperarousal, and negative alterations in mood (American Psychiatric Association, 2022). Childbirth-related posttraumatic stress symptoms (CB-PTSS) are distressing and can impair one’s capacity to engage in certain activities (Ayers et al., 2018), such as infant care. In addition, approximately 22% of women report having already sought treatment for these symptoms and 16% express a wish to receive appropriate treatment (Ayers et al., 2018). CB-PTSS may also increase vulnerability to other mental health disorders such as postpartum depression and anxiety (Dekel et al., 2020; Dikmen-Yildiz et al., 2017). Notably, traumatic childbirth is a distinct traumatic experience, as it has several specific characteristics, including being socially perceived as a positive event and occurring during an important life transition (Horesh et al., 2021). Traumatic childbirth experiences and CB-PTSS can profoundly affect women’s daily functioning and wellbeing (Ayers et al., 2018), as well as their relationships with their child and partner (e.g., Van Sieleghem et al., 2022; Garthus-Niegel et al., 2018).

Women with CB-PTSS tend to report more incoherent, emotionally intense and sensory-based memories of their childbirth experience, which are more frequently recalled and re-experienced as childbirth-related intrusive memories (CB-IM) (Thiel et al., 2021). Approximately a quarter of women report CB-IM at 6 weeks postpartum (Briddon et al., 2015), which can persist for several years (Deforges et al., 2022). These symptoms, often referred to as “flashbacks” by non-specialists, are most often experienced as visual images or short video-like sequences, although they can occur in any sensorial modality (Fort et al., 2026). For example, women may involuntarily relive the pain of contractions, specific medical procedures such as emergency caesarean section, or distressing interpersonal interactions with healthcare professionals (Fort et al., 2026).

Among CB-PTSS, CB-IM appear to play a central role in symptomatology. They are both one of the most frequently observed symptoms following birth trauma (Delicate et al., 2022) and a theorised driver of broader symptom development and maintenance (McNally, 2012), including, but not limited to, ruminative thinking (Evans et al., 2007). Certain characteristics of IM have been associated with greater PTSS severity, particularly the feeling that the event is happening again in the present moment (“nowness”) and the distress level they elicit (Evans et al., 2007; Michael et al., 2005), although such associations have not consistently been observed for CB-IM (Fort et al., 2026).

Notwithstanding the significant burden associated with CB-PTSS, evidence-based treatments specifically tailored for this population [i.e., trauma-focused cognitive behavioural therapy and eye movement desensitization and reprocessing (EMDR)], which are recommended by the World Health Organization (WHO) (World Health Organization, 2013) and the National Institute for Health and Care Excellence (NICE) (National Institute for Health and Care Excellence, 2018), remain limited (de Bruijn et al., 2020). Therefore brief, scalable, and cost-effective interventions capable of reaching the large number of women affected are required (Jomeen et al., 2025). Given the proposed role of CB-IM in the development and maintenance of CB-PTSS, recent work has focused on interventions that directly target these symptoms. Grounded in cognitive models suggesting that IM arise from heightened encoding of sensory, particularly visual, information related to traumatic experiences (Brewin and Holmes, 2003), a single-session intervention aimed at interfering with the visual components of IM through memory-updating processes has been developed (Kessler et al., 2018).

This behavioural intervention involving a visuospatial task (BI-VT), involves two main components. First, participants are asked to retrieve the traumatic memory, typically by briefly narrating the event. This step is intended to destabilise the memory, making it temporarily labile and susceptible to modification (Lee et al., 2017). Participants are then instructed to engage in a visuospatial task, which is assumed to compete for the working memory resources required to restabilise the memory during the memory reconsolidation process (Meir Drexler and Wolf, 2018). By interfering with this process, the memory is expected to become less likely to intrude involuntarily (Meir Drexler and Wolf, 2018).

Using the videogame Tetris as a visuospatial task, this approach has received strong support in an experimental setting, showing a large reduction in IM (Varma et al., 2024). The first clinical application of a BI-VT was reported by in an open-label single case series with inpatients presenting complex PTSD (Kessler et al., 2018) and was followed by single-arm studies in diverse clinical populations, all reporting reductions in IM (Deforges et al., 2022; Kanstrup et al., 2021; Singh et al., 2021; Thorarinsdottir et al., 2021; Thorarinsdottir et al., 2022; Hardarson et al., 2024; Kubickova et al., 2024; Lau-Zhu and Chan, 2025). Four subsequent randomised controlled trials (RCT) conducted with health-care professionals with work-related trauma found significant reductions in IM following this intervention, compared with delayed intervention arms (i.e., a reduction of 78% in IM number was reported between pre- and post-intervention) (Iyadurai et al., 2023; Ramineni et al., 2023), or active control conditions (i.e., a reduction of 86% in IM number between pre- and post-intervention, and 72% fewer IM in the intervention group compared to a control group were reported) (Kanstrup et al., 2024; Beckenstrom et al., 2026). However, one RCT reported no significant differences between conditions (i.e., BI-VT versus a verbal condition involving reading an online article and answering questions) in PTSD patients (Kehyayan et al., 2024), although this may be partly attributable to crossover trial design limitations.

A BI-VT was first tested in women reporting established CB-IM after traumatic childbirth in a single-group pre-post pilot study (*n* = 18), which showed a median reduction of 82% in the number of CB-IM, and a mean reduction of 57% in overall CB-PTSS severity, supporting its feasibility and preliminary efficacy (Deforges et al., 2022). Of note, in this BI-VT, returning to the original traumatic context (i.e., the maternity ward where the birth took place) was used during the traumatic memory recall phase in addition to childbirth narrative to facilitate memory reactivation by exposure to potential retrieval cues (Deforges et al., 2022).

However, beyond symptom reduction, it is essential to understand how such interventions are subjectively experienced in order to assess their acceptability and better identify their underlying mechanisms (Timulak and Keogh, 2017). This is particularly relevant for BI-VT, for which the mechanisms of action remain under debate (e.g., Lattal, 2024).

In the broader trauma literature, a qualitative synthesis indicates that trauma-focused treatments can be experienced as emotionally demanding, yet patients often perceived the processing of these emotions as a necessary component of recovery (Gjerstad et al., 2024; Lepisto et al., 2025). Similar processes may be engaged in the present BI-VT, which involves both returning to the maternity ward and briefly narrating the traumatic childbirth experience, components that may elicit distress while facilitating therapeutic change.

Qualitative approaches can also reveal helpful or hindering processes that were not initially theorised. For instance, patients frequently emphasize the importance of non-specific therapeutic factors, such as a supportive therapeutic relationship and a safe therapeutic environment, alongside treatment-specific components related to trauma-focused treatments like the processing of traumatic memories recovery (Gjerstad et al., 2024; Lepisto et al., 2025). Understanding which mechanisms patients identify as driving change is therefore critical for refining both the interventions and their theoretical foundations (Timulak and Keogh, 2017; Salkovskis et al., 2023).

These considerations are particularly relevant for the single-session BI-VT targeting CB-IM examined in the present study, which combines two steps: recalling the traumatic childbirth memory (including return to the trauma context and brief narration of the traumatic childbirth experience) and playing Tetris. The respective contributions of these steps to therapeutic change, as well as how they are subjectively experienced, remain unknown. To date, no study has specifically examined how women experience this intervention or how they interpret its effects.

The present qualitative study explored: (1) how participants experienced the two steps of a single-session BI-VT targeting CB-IM (recalling the traumatic childbirth memory and playing Tetris); (2) what changes participants perceived in their CB-PTSS and daily life following the intervention; and (3) what mechanisms participants believed might explain these changes.

## 2 Materials and methods

### 2.1 Study population

The present qualitative study was embedded within a larger multicentric, single-blind, waitlist-controlled randomised trial examining the effects of an intervention on CB-IM, whose protocol was pre-registered on ClinicalTrials.gov (NCT05381155) and published (Fort et al., 2023). The RCT received ethical approval from Human Research Ethics Committee of the Canton de Vaud (study number 2022-00652).

Participants were mothers aged ≥18 years who had given birth at least 6 weeks prior to screening in one of two Swiss study centres (i.e., maternity wards of a university hospital and a regional hospital) and reported CB-IM. To minimise floor effects, inclusion required at least four CB-IM in the 2 weeks prior to screening. Main exclusion criteria were insufficient French proficiency, intellectual disability or psychotic disorder, or ongoing psychological treatment related to childbirth. Full eligibility criteria are reported in the published protocol (Fort et al., 2023). Women who had recently given birth in either of the two study centres received text messages to inform them about the study. In addition, advertisements were placed in locations frequently visited by mothers, such as private gynaecology and paediatric practices, midwifery offices, nurseries, parent associations, and on social media. If interested, women could directly contact the research team via the contact details provided on the flyer, or complete an online form on a dedicated study website. A first screening phone call was organised with a trained psychologist who presented the study, obtained oral consent, and assessed women’s eligibility. If eligible, women received the information sheet for review prior to providing written informed consent and being enrolled in the study.

A subsample of participants from the immediate intervention arm (*n* = 59) who had provided specific consent to take part in a qualitative interview at the time of RCT enrolment was invited to participate in semi-structured interviews. Participants from the waitlist control arm (*n* = 61) were not included, as the aim was to capture experiences of the intervention as delivered under standard conditions, without the potential influence of delayed access to the intervention. The subsample of participants was recruited using a maximum variation sampling strategy based on self-reported change in CB-IM number at the end of the RCT procedures using a single question: “*Since the intervention, has the number of CB-IM you experienced increased, diminished or remained unchanged on a scale from -10 to 10? -10 meaning that the CB-IM number has extremely diminished, 10 that it has extremely increased, and 0 that it is unchanged*.” Three groups were defined: improvement (score −10 to −3), little or no change (−2 to +2), and worsening (+3 to +10). Of the 46 participants who completed the RCT quantitative procedures in the immediate intervention arm and responded to this screening question, 44 reported improvement, one reported little or no change, and one reported worsening. Consequently, the recruitment target for participants reporting improvement was reached rapidly (targeted *n* = 7), while only one eligible participant was available in each of the other two groups. The research team therefore decided to include additional participants reporting improvement.

**Figure 1:**
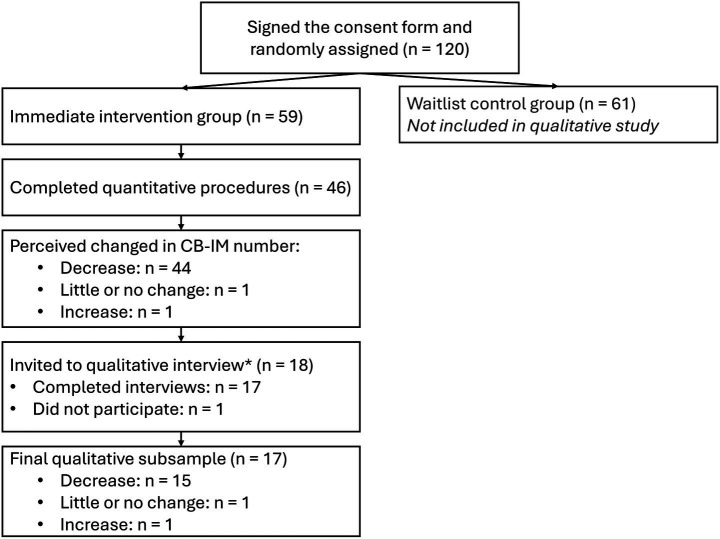
Participant flow diagram for the qualitative subsample. CB-IM: Childbirth-related intrusive memories. *According to the maximum variation sampling strategy utilised.

Participants were invited to take part in the qualitative interview during a phone call by a research team member who administered the question related to the evolution of CB-IM number used for sampling purposes, but who was not involved in the delivery of the intervention. Of the 18 participants invited, one did not complete the interview following initial contact with no reason mentioned, resulting in a final sample of 17 participants (*n* = 15 improvement, *n* = 1 little or no change, *n* = 1 worsening) (see Figure 1).

### 2.2 Intervention-related procedures

**Figure 2:**
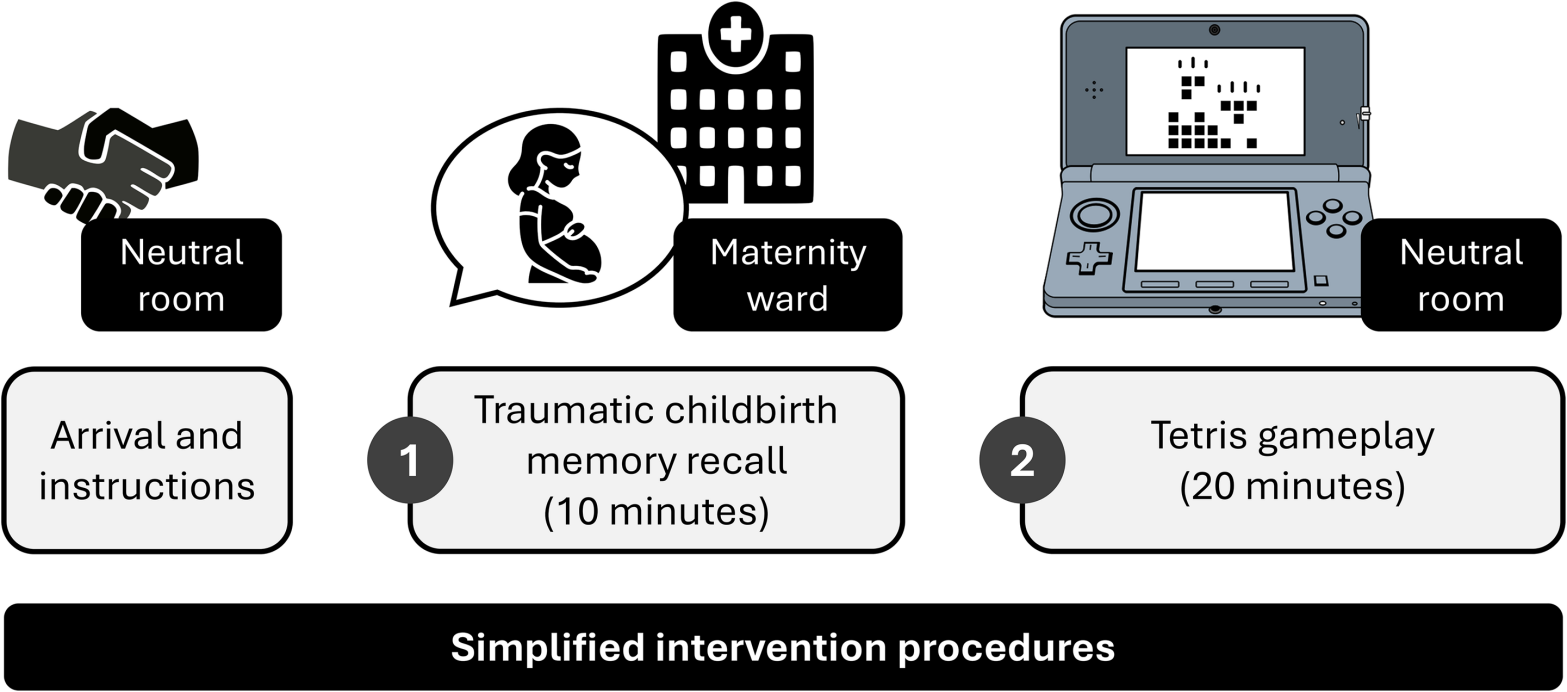
Intervention procedures. CB-IM, Childbirth-related intrusive memories. Icons from the Noun Project (CC BY 3.0): Pregnancy by PictoCraft Studios, and Tetris by Andi Nur Abdillah. Remaining illustrations are public domain, retrieved from OpenClipart.

Intervention-related procedures encompass both a pre-intervention CB-IM monitoring phase and the intervention itself, see Figure 2 for a visual representation.

Prior to the intervention, participants noted down their CB-IM over a two-week period using a structured daily diary (i.e., a CB-IM log). Each time they experienced a CB-IM, they were asked to briefly write down its content and sensorial modalities, as well as rate the level of distress elicited and its sense of nowness. Importantly, this diary was not intended to function as a detailed trauma narrative; rather, it served as a brief, momentary record of CB-IM.

Participants were first welcomed by the interviewer in a neutral building (i.e., administrative building) to receive instructions about the intervention procedures. For the traumatic childbirth memory recall, participants went with the interviewer to the maternity ward where they had given birth to briefly narrate their childbirth experience in an examination room for about 10 min. Participants were asked to first narrate their overall childbirth experience for five to 7 min, and during the remaining time (3 to 5 min), to describe in more detail the moment associated with their most frequent and/or distressing CB-IM that they had reported in their daily diary for the last 2 weeks. Participants then returned to the neutral building to perform the visuospatial task involving the videogame Tetris for 20 min (see Fort et al., 2023 for detailed instructions). The intervention lasted for approximately 1 h. During the whole intervention procedure, participants were accompanied by a trained psychologist to give them instructions, help them manage their potential distress, and to encourage compliance.

Importantly, prior to receiving the intervention, participants were not informed about its specific components in order to minimise expectation bias and to capture their naïve impressions of the mechanisms involved. They were only told that the procedure consisted of a brief narration of their childbirth experience combined with a visuospatial task, without being informed that this involved returning to the maternity ward and playing Tetris. Only after completing the intervention participants were debriefed and informed about its underlying scientific rationale.

### 2.3 Data collection

Semi-structured interviews were conducted by the investigator who had delivered the intervention during the RCT (DF) following completion of the post-intervention quantitative assessments, ranging from 45 to 77 days after receiving the intervention (between December 2022 and March 2025). Interviews were guided by a semi-structured interview guide, which was developed for this study and was not pilot tested (see Table 1). Participants were reminded at the outset of the interview that there were no right or wrong answers and that their honest perceptions were sought.

**Table 1:** Interview guide.

Interview questions
How did you feel about reporting your flashbacks^a^ in the diary^b^?
How did flashbacks generally manifest themselves before our appointment?
What was your experience of the appointment at the maternity? – What can you tell about the experience about going back there? Additional questions if needed: How did you feel about going back to the maternity ward and telling the story of your birth? What emotions did you feel when you went to the maternity ward and how did you cope with these emotions?
Why do you think we went to the maternity ward on our meeting?
And why do you think I asked you to narrate your childbirth there?
What did you think, what did you feel when you found out that you were going to play Tetris after that? Additional questions if needed: What was it like for you to play it for 20 min? How well did you manage to concentrate on the game? – can you tell me about you concentrate how did you concentrate to play the game?
What do you think Tetris was used for?
What changes, if any, did you observe after the appointment?
What changes, if any, did you observe regarding your flashbacks after the appointment? Additional questions if needed: How do flashbacks generally manifest themselves since our appointment? What, if any, changes have you observed in the characteristics of your flashbacks?
What changes have you observed since our meeting regarding your psychological well-being? Additional questions if needed: What changes, if any, have you noticed since our appointment about avoiding things or people that might make you think about childbirth? What changes, if any, have you observed in relation to any negative thoughts or moods you may have in general since our appointment? Since our appointment, what changes, if any, have you noticed about your feeling of being easily irritable or under stress?
How has the significance of your childbirth experience changed since our appointment?
What changes, if any, have you observed in your daily life since the appointment?
Please tell me if there have been any changes in your relationship with your child or children since our last appointment?
Please tell me if there have been any changes in your relationship with your partner since our last appointment?
In your opinion, what do you think explains the changes you mentioned?

a The term “flashbacks” refers to childbirth-related intrusive memories, as it is more widely recognised and used by non-specialists.

b The term “diary” refers to a structured daily log in which participants briefly recorded their childbirth-related intrusive memories; it was not intended as a detailed narrative account.

Interviews were conducted in French via videoconference to accommodate the scheduling constraints of the study population. All interviews were audio-recorded, transcribed using Amberscript B.V. (2025), and subsequently verified for accuracy. Interviews lasted on average 41 min (range: 20–75 min). Excerpts presented in this article were translated into English by the research team.

### 2.4 Data analysis

Interviews were analysed using inductive content analysis following the approach described by Bengtsson (Bengtsson, 2016). This approach involves four steps: decontextualization, recontextualization, categorization, and compilation. First, the transcripts were read in their entirety to gain an overall sense of the data. Meaning units relevant to the research question were then identified and condensed into codes. Codes were subsequently grouped into subcategories and categories based on their similarities and differences. Finally, categories were reviewed in relation to the full dataset to ensure they accurately represented the data.

All 17 interviews were coded independently by two coders (DF and ES), a psychology PhD candidate and a nursing sciences PhD, using MAXQDA Standard 2022 software from VERBI GmbH, Berlin. One coder had been involved in delivering the intervention and conducting the interviews, providing in-depth familiarity with the data (DF), while the second coder was not involved in data collection (ES), providing an independent analytical perspective. This combination of perspectives constitutes a form of investigator triangulation, enhancing the credibility of the analysis (Patton, 2015). Disagreements between coders were resolved through discussion until consensus was reached. Throughout the data collection and analysis process, the interviewer and coders maintained a reflexivity journal to document their impressions and analytical decisions, supporting transparency and reflexivity in the research process. The coders consisted of two female researchers with expertise in perinatal mental health and experience in conducting qualitative research, but without personal experience of traumatic childbirth. The interviewer also delivered the intervention, creating a pre-existing therapeutic relationship. Awareness of this positionality and its potential influence on both data collection and analysis was central to the reflexive process. The reflexivity journal maintained throughout data collection supported ongoing critical reflection on how this relationship may have shaped participants’ disclosures and the analytical process.

Data saturation was not formally assessed in this study. The qualitative analysis was conducted on a predefined subsample derived from an RCT, which limited the possibility of iterative, data-driven sampling. Consequently, the aim was not to achieve theoretical saturation, but rather to explore in depth the experiences of participants within this subsample. The analysis was conducted systematically to capture the range and diversity of perspectives present in the data. Transcripts and study findings were not returned to participants for comment or correction, as the study design did not include procedures for participant validation.

## 3 Results

Participants’ ages ranged from 30 to 46 years. The majority were of Swiss nationality (*n* = 10/17), had a tertiary level of education (*n* = 10), and were married or cohabiting (*n* = 13). In terms of obstetric characteristics, time since childbirth ranged from 4 months to 12 years and 11 months. Childbirth modes were diverse: most participants had vaginal births (*n* = 10), while other modes were also represented, including emergency caesarean sections (*n* = 5), elective caesarean sections (*n* = 3), and instrumental births (vacuum or forceps-assisted; *n* = 2). Gestational age at birth ranged from 28 to 41 weeks, and birth weights varied from 1,500 to 4,330 grams. A minority of participants reported already having received a prior treatment related to their traumatic childbirth experience (*n* = 4). Detailed individual characteristics are presented in Table 2.

**Table 2:** Sample characteristics (*n* = 17).

ID	Age	Nationality	Education level	Marital status	Time since childbirth	Childbirth mode	Prior treatment	Perceived change in CB-IM number
P01	45	Swiss	Tertiary	Partnered	12y 11 m	Vaginal birth	No	−10
P02	37	Swiss	Secondary	Partnered	6 m	Vacuum-assisted birth	No	−10
P03	36	French	Tertiary	Partnered	1y 7 m	Vaginal birth	Yes	−8
P04	45	Swiss	Secondary	Partnered	1y 2 m	Elective c-section	No	+10
P05	32	Peruvian	Tertiary	Single	9 m	Vaginal birth	No	−10
P06	34	French	Tertiary	Partnered	1y	Vaginal birth	Yes	−9
P07	35	Swiss	Tertiary	Separated	5 m	Vaginal birth	No	−5
P08	41	English	Tertiary	Partnered	2y 1 m	Emergency c-section	No	−9
P09	38	Peruvian	Tertiary	Partnered	1y	Emergency c-section	No	0
P10	37	Swiss	Secondary	Partnered	5 m	Emergency c-section	No	−9
P11	46	Swiss	Tertiary	Single	3y 6 m	Elective c-section	No	−5
P12	35	Swiss	Secondary	Separated	2y 10 m	Emergency c-section	No	−9
P13	35	Swiss	Secondary	Partnered	4 m	Forceps-assisted birth	No	−5
P14	39	English	Tertiary	Partnered	11y 8 m	Vaginal birth	Yes	−9
P15	35	Swiss	Primary	Partnered	7 m	Vaginal birth	No	−5
P16	30	French	Tertiary	Partnered	4 m	Elective c-section	No	−7
P17	37	Swiss	Secondary	Partnered	1y 9 m	Emergency c-section	Yes	−6

Perceived change in CB-IM number was assessed using a self-reported scale ranging from −10 (extreme decrease) to +10 (extreme increase), as described in Materials and Methods. Categories were defined as improvement (−10 to −3), little or no change (−2 to +2), and worsening (+3 to +10).

### 3.1 Experience of the intervention

The experience of the intervention is divided between *recalling the traumatic childbirth memory* and *playing Tetris*. These two themes are presented below and in Table 3.

**Table 3:** Experience of the intervention.

Main category	Category	Subcategory	n
1. Recalling the traumatic childbirth memory	Challenging aspects	Stress	8
		Sadness	8
		Destabilisation	7
		Difficult	7
		Anger	5
		Fear	3
		Guilt	3
		Frustration	1
		Triggered CB-IM	1
	Positive aspects	Moving	6
		Positive emotions	6
		Curiosity	2
		Nostalgia	1
		Surprise	1
		Excitement	1
	Coping strategies	Allowing the expression of one’s emotions	6
		Presence of the interviewer	6
		Taking a step back	2
		Letting emotions pass	1
		Focusing on the positive	1
2. Playing Tetris	Initial reactions	Surprise	7
		Doubtful	4
		Indifference	3
	Engagement in the game	Challenging/exciting	14
		Soothing/joyful	12
		Tedious/tiresome	2
		Focusing	1

#### 3.1.1 Recalling the traumatic childbirth memory

Recalling the traumatic childbirth memory was characterised by two contrasting patterns: a predominantly intense and difficult emotional experience on the one hand, and, for many participants, a simultaneously moving and positive dimension on the other. For several participants, these two patterns coexisted, reflecting the complexity and ambivalence of returning to the maternity ward where the traumatic childbirth happened and narrating it. Notably, for an important proportion of participants (*n* = 10), the intervention also represented their first time returning to the maternity ward since childbirth, adding an additional layer of emotional salience to the process.

Participants reported a range of negative emotions during this phase, including stress (*n* = 8), sadness (*n* = 8), destabilization (*n* = 7), and described the experience as simply difficult (*n* = 7).

*“I wasn’t feeling well at the time. […] Clearly, I was under a lot of stress when we were at the maternity ward. I felt hot, my heart was beating much faster, and I was, well, I wasn’t feeling well.”* (P02)

Anger in relation to what happened during childbirth (*n* = 5) and fear (*n* = 3) were also reported. A smaller number of participants mentioned feelings of guilt against oneself for what happened during childbirth (*n* = 2), and one participant reported that returning to the maternity ward triggered CB-IM.

In response to these intense emotions, several participants described strategies they mobilized when returning to the maternity ward to cope with their emotions. Allowing the expression of one’s emotions was the most frequently reported strategy (*n* = 6), with some participants also describing taking a step back (*n* = 2) or other individual strategies.

*“I just let it come. Really let it come. And then… that was it. I try not to hold things in anymore. I mean, if something is felt, there’s a reason for it. So, it’s about letting it come, letting it out.”* (P13)

Notably, six participants also mentioned that the presence of the interviewer helped them feel at ease and supported when recalling their childbirth.

*“Well, I cried a bit, and then I was there… I mean, I was there, and you were there too. And that, that helped me feel… you know, supported. So, it was okay.”* (P12)

Despite this emotional intensity, a significant number of participants also described the experience as moving (*n* = 6) and reported positive emotions (*n* = 6).

*“It was still […] kind of an exciting moment, a bit of a desire to go back, a sense of nostalgia… well, I don’t know, it’s a bit of a strange feeling. Also some curiosity. As if it were a kind of sacred place.”* (P01)

*“It was quite moving […] I really didn’t expect to have these—how can I put it—these reactions, or rather these sensory triggers. The colours, and then the colours, the smells, all of that.”* (P09)

Interestingly, one participant noted that allowing herself to experience these negative emotions subsequently enabled her to access positive emotions that had previously been overshadowed.

*“At first, I felt fear and apprehension, but then afterwards, I experienced a lot of these negative emotions, which eventually gave way to positive ones, where suddenly I could clearly remember giving my son his first bath in the maternity ward […] I also remembered […] moments when I held my son in my arms for the first time. In fact, pleasant moments that I had not really been able to access […] Then, after processing these negative experiences, positive things were able to come back. In fact, it was also a moment in the maternity ward that was actually good.”* (P12)

#### 3.1.2 Playing Tetris

Reactions to playing Tetris following the return to the neutral administrative building were characterized by a temporal progression: from initial surprise and scepticism to engagement and challenge during the game, to a predominant sense of relief and calm.

Upon learning they would be playing Tetris, many participants expressed surprise (*n* = 7), while others were doubtful about the relevance of the task (*n* = 4) or reported indifference (*n* = 3).

*“Well, I thought to myself: ‘What does playing Tetris have to do with the flashbacks I’m having? How can a game help me, uh, feel better?’”* (P02)

*“So, it made me laugh. I really wasn’t expecting that.”* (P10)

Once engaged in the game, the experience was described as positively challenging and exciting, with 14 of 17 participants reporting this reaction.

*“It’s true that it’s a somewhat challenging game, and then it becomes almost addictive after a while, I feel—you want to go faster, to succeed, well, that’s it.”* (P01)

This engagement was followed for most participants by a sense of calm and relief, with 12 participants describing the experience as soothing or joyful.

*“For me, it […] really helped me relax. […] it was kind of a comfort for me. So, I had a sort of safe place where I could retreat. It was very good.”* (P10)

Two participants reported finding the game tedious or tiresome.

*“At the time, it annoyed me because I don’t like video games [laugh]. And then I thought: ‘Oh my! I’m going to have to learn something new […]’ And then I thought it was going to take longer […] and in the end, I was able to get through it quickly.”* (P01)

*“After a while, I mean… 20 minutes felt quite long, and at the beginning there was more excitement, but when I actually had to play the game, I was like, “Oh no, this is kind of annoying!” I got a bit bored pretty quickly… having to play a video game for that long. I guess I’m just not used to playing.”* (P11)

### 3.2 Perceived changes following the intervention

Perceived changes following the intervention are related to CB-IM, the relationship to the childbirth memory, avoidance, negative mood, arousal, family relationships, and other perceived changes. A summary of the coding tree is presented in Table 4.

**Table 4:** Perceived changes following the intervention.

Main category	Category	Subcategory	n
1. Changes in CB-IM	Number	Decrease	15
		No change	1
		Transient increase	1
	Emotional intensity^a^	Decrease	12
		No change	3
	Nowness^a^	Decrease	8
		No change	7
	Content^a^	No change	9
		Reduction in variety of content	6
	Sensorial modalities^a^	No change	10
		Modification of sensory modalities	5
	Other changes^a^	Shorter	4
		Less unpredictable	2
		Less involuntary	2
2. Changes in the appraisal of the childbirth memory		Acceptance of the childbirth memory	9
		Greater control over the memory	7
		Putting the childbirth into perspective	6
		Freed from a burden	5
		More positive appraisal of the childbirth	4
		Recalling memories associated with childbirth	4
		Becoming aware of the impact of the childbirth	4
		Fewer thoughts about childbirth	3
3. Changes in avoidance, mood, and arousal symptoms	Avoidance	No prior symptoms	10
		Decrease	5
		No change	2
	Negative mood	Decrease	11
		No change	4
		No prior symptoms	2
	Hypervigilance	No prior symptoms	6
		Decrease	6
		No change	5
	Irritability	No change	8
		Decrease	4
		No prior symptoms	3
		Increase	2
4. Changes in relationship with child and partner	Relationship with child	No change	13
		Feeling less guilty	1
		Feeling more at ease with child	1
		Feeling more attentive toward child	1
		Child perceived as more settled	1
	Relationship with partner^b^	No change	10
		Feeling closer to partner	3
5. Additional perceived changes		No additional changes	9
		Traumatic childbirth no longer represents a barrier for considering a new pregnancy	5
		Improvement in sleep quality	4
		Increased energy	3
		Fewer nightmares	2
		Sense of serenity	2
		Transient adverse effects on sleep	1

a Excludes two participants who reported no longer experiencing CB-IM following the intervention.

b Excludes four participants that were single or separated.

#### 3.2.1 Perceived changes in CB-IM

Perceived changes in CB-IM following the intervention were reported at different levels: a perceived reduction in their frequency and emotional intensity, and qualitative modifications in the way memories were experienced.

The most striking finding was the reduction in the number of CB-IM reported by 15 of 17 participants.

*“And now I even have the impression that they are, that they have disappeared.”* (P13)

*“A decrease, even a complete disappearance of the flashbacks, because very honestly I haven’t had any since [the intervention].”* (P06)

Of note, the marked reduction in CB-IM number reported by most participants was sometimes striking enough to prompt active verification: two participants reported deliberately attempting to trigger CB-IM after the intervention, surprised by their reduction following the intervention.

*“Well, I don’t have any flashbacks at all anymore. And even if I try to bring them back, I can’t manage to have them.”* (P01)

One participant reported no change, and one reported a transient increase in CB-IM number that subsequently resolved to their initial level.

*“It was amplified with the appointment, the fact […] of having to recall that moment, it became something quite intense, yes. […] I feel like we’ve gone back to before the diary. In the sense that […] it has become again something that is part of everyday life, of routine. They are there […] and they are not there every day. […] I feel like I’ve returned, in terms of frequency, somewhat to the starting point where I felt I had a few of them.”* (P04)

Beyond the number of CB-IM experienced, their emotional intensity also decreased markedly, with 12 participants reporting that their CB-IM felt less emotionally overwhelming than before the intervention. The remaining three participants reported stability in emotional intensity. It should be noted that two participants reported a complete absence of CB-IM following the intervention and were thus not included in the description of characteristics associated with CB-IM, as they could not evaluate such changes.

*“It will generate a small negative feeling in me for a short period of time, but it won’t affect the whole day anymore, let’s say, as it could do at the beginning.”* (P10)

*“[The flashback] is not experienced as traumatic. That is to say, if I have a flashback of the operating room, even now, even if I have to talk about very clear images and physical sensations […], I don’t experience it anymore, I don’t feel it in my body anymore; I no longer have the emotional burden to talk about it as I used to. I think I have a more coherent and more accurate account of the experience […] So, […] they are no longer really flashbacks, I think, they are memories.”* (P06)

Further qualitative changes in the nature of CB-IM were also reported. Notably, eight participants described a reduction in the sense of nowness.

*“I remember things related to my childbirth, but now at home or elsewhere there is a certain distance; I realise that it is not something I am currently experiencing.”* (P09)

The content of CB-IM remained largely stable for most participants (*n* = 9), while six participants reported a narrowing of the content of CB-IM, with some scenes or elements becoming less present (*n* = 6). Several participants also reported additional qualitative changes, including shorter CB-IM (*n* = 4), and modifications in sensory modalities (*n* = 5). A smaller number described their CB-IM as less unpredictable (*n* = 2) or less involuntary (*n* = 2).

*“I only have an image. It’s not like a film I’m inside of, playing out; it’s more like one or two images.”* (P14)

*“And it is often linked to something—so it remains involuntary—but it may be linked to… I don’t know, a song, or a very specific conversation. So, it is more tied to a particular event. […] It is less complete. It is shorter. […] Before, I would really have images each time—of myself, of the baby. It was like an image, as if it were taken from above, from a high angle, where we could be seen at that moment. And that is what I was seeing. I would see the scene either in motion or broken up into sequences. And now I would say that it is… it is less of a whole scene, and more of a very brief, specific moment.”* (P05)

#### 3.2.2 Changes in the appraisal of the childbirth memory

Beyond changes in CB-IM, participants reported broader shifts into the appraisal of the childbirth memory itself, characterized by a greater sense of acceptance and control over the memory, as well as a deeper transformation in how the childbirth experience was perceived and integrated.

The most prominent change was an increased acceptance of the childbirth memory, reported by nine participants. Closely related, seven participants described feeling more in control of the memory, and six reported being able to put the childbirth experience into perspective.

*“I manage […] to tell myself that it was a childbirth, and that alone is positive. Because before, I used to say it was an operation; I didn’t feel like I had given birth. […] Whereas before it was really just mechanical. It was an operation, it was really only linked to everything negative: hospital, operation, blood, pain. Whereas today I can tell myself no, it was a childbirth, it was my way of delivering my baby who is now healthy.”* (P16)

*“It is like a memory. It is stored among all the other memories I have of my past now. It is no longer the drawer that triggers an atomic bomb in my body.”* (P06).

Several participants also described a sense of being freed from a burden (*n* = 5), suggesting that the intervention facilitated not only cognitive but also emotional processing of the childbirth experience.

*“I felt a kind of soothing effect, as if I had […] been freed from a burden. And then… yes, it made me… Afterwards, I really felt calm. So, I really feel that, in the end, it was very positive to do all of this.”* (P12)

A more positive appraisal of the childbirth memory was reported by four participants, and four others described recalling memories of childbirth that had previously been inaccessible.

*“But it remains, therefore, a significant memory but not a traumatic one. It is now more of a positive memory, even today.”* (P06)

*“I have made peace with [my childbirth]. In any case, I can’t change anything. […] The good things, actually. There are many of them. It is… well, it is still an incredible experience to see my son for the first time. All these things that are very moving. So, I really try to hold on to them. And then of course, there are still small moments where there is still this slightly bitter taste, but well, that’s it.”* (P09)

Four participants also reported becoming more aware of the impact the childbirth had had on them, suggesting that for some, the intervention prompted a process of conscious recognition of their trauma. A smaller number reported fewer thoughts about the childbirth overall (n = 3).

*“In fact, I didn’t expect that it would make me realise that I was actually having a lot of flashbacks. And I also didn’t think that my childbirth affected me that much, especially by going back to the maternity ward.”* (P07)

#### 3.2.3 Changes in avoidance, mood, and arousal symptoms

In addition to changes in CB-IM reported above, participants were asked about perceived changes across three further DSM-5 posttraumatic stress symptom clusters: avoidance, negative alterations in mood and cognition, and alterations in arousal and reactivity.

Regarding avoidance, most participants (*n* = 10) reported no avoidance symptoms prior to the intervention. Among the seven participants who did report avoidance, five described a reduction following the intervention, while two reported no change.

*“I prepared for my childbirth with music […] And I remember that when we were in the delivery room, […] I often asked my partner to put the music on to help me cope a bit and… And afterwards, I didn’t want to hear that music anymore, it brought back too many bad memories […]. And now, little by little—but this has been very gradual—I can listen to it again, and I don’t start crying or try to skip it anymore.”* (P05)

Negative mood showed the most consistent improvements across the sample. Only two participants reported no negative mood symptoms prior to the intervention. Of the remaining 15, eleven reported a reduction in negative mood following the intervention, while four reported no change.

*“I feel like I’ve had many more joyful moments. […] In fact, I tend to kind of bounce around a bit when I’m happy. And I find myself being a bit like I was before. Since I had my son, I’ve almost felt like I became a very serious mother. And now I’m kind of reliving how I used to be before. […] And I feel a bit more like that now, ready to play.”* (P14)

Changes in arousal and reactivity symptoms were more variable. Regarding hypervigilance, six participants reported no symptoms prior to the intervention. Of the eleven who did, six reported a reduction, four reported no change, and one reported an increase. Irritability showed the least change of all symptom clusters: three participants reported no prior symptoms, and of the fourteen who did, only four reported a reduction, while eight reported no change and two reported an increase following the intervention.

*“I have to admit that I had quite a bit of hypervigilance towards my son in the first months, and I can see that it is gradually decreasing. […] And now, over the months, I see that it is getting a bit better, let’s say… it’s easier to let him do things, to stay behind him. He also has his little moments on his own, when I’m not always watching everything he does. After the appointment, I had the feeling of being much more at ease, let’s say, of having less fear that something might happen to him.”* (P10)

*“But it’s true that I am more irritable than usual. But then again, there are many factors, you have children…”* (P04)

#### 3.2.4 Changes in relationships with child and partner

Participants were also asked about perceived changes in their relationships with their child and partner. Regarding the relationship with their child, most participants reported no change (*n* = 13). Four participants described relational improvements, including feeling less guilty toward their child (*n* = 1), perceiving their child as more settled (*n* = 1), feeling more at ease with their child (*n* = 1), and feeling more attentive toward their child (*n* = 1).

Regarding the relationship with their partner, four participants were single and therefore not asked this question. Of the 13 remaining participants, the majority reported no change (*n* = 9), while four reported positive changes, including three who described feeling closer to their partner following the intervention.

*“Well. I think I… but maybe I am less so now, as I was explaining that I had this physical hypersensitivity. I still had quite disproportionate reactions to certain things. I feel like I don’t really have them anymore these days, so it is much calmer. I feel like we have also reconnected more like before since the interview, at least over these past weeks, whereas I was still not at all comfortable in my body and not at all comfortable with my emotions.”* (P06)

#### 3.2.5 Additional perceived changes

When asked whether they had noticed any other changes following the intervention, nine participants reported no additional changes beyond those already described. Eight participants spontaneously reported additional benefits, including improvements in sleep quality (*n* = 4), increased energy levels (*n* = 3), fewer nightmares (*n* = 2), and a sense of serenity (*n* = 2). One participant reported transient adverse effects in the days following the intervention, including disturbed sleep and drowsiness, which resolved subsequently.

Finally, five participants reported that their traumatic childbirth experience no longer represented a barrier to considering another pregnancy.

*“There is one aspect where I can see a real change: before the appointment, talking about the possibility of having a second child with my husband was… it was… I couldn’t talk about it. […] There has been a shift. Now I am more able to pause and tell myself, okay, there may be… not right away, but a possibility that in the future we could have another child. And that is really where I see a quite clear change.”* (P10)

### 3.3 Perceived mechanisms of change

When asked about the perceived mechanisms underlying the changes they had experienced, most participants attributed these changes either solely to the intervention (*n* = 7) or to a combination of intervention-related and external factors (*n* = 7). Three participants attributed changes exclusively to factors external to the intervention, such as life events or the passage of time. Table 5 presents a summary of codes attributed in relation to perceived mechanisms of change.

**Table 5:** Perceived mechanisms of change.

Category	Subcategory	n
1. Intervention-related mechanisms	Talking/writing about the childbirth experience	10
	Combination of childbirth narrative and Tetris	6
	Returning to the maternity ward	5
	Processing the traumatic childbirth experience	2
2. External factors	Concurrent life changes	6
	Passage of time	5
	Other therapeutic approaches	2

Seven participants attributed changes solely to the intervention, seven participants attributed changes to a combination of intervention-related mechanisms and external factors, three participants attributed changes solely to external factors.

Among intervention-related mechanisms, talking about and writing down CB-IM was the most frequently reported mechanism, mentioned by ten participants. For these participants, the act of verbalizing or externalizing their experience appeared to be a key therapeutic element of the intervention.

*“Externalising it and then maybe re-analysing it afterwards, in a ‘cold’ way, let’s say.”* (P10)

*“Maybe the fact of verbalizing them, of writing them down, of… yes, realizing that something came up and that it is not insignificant. And also, yes, not necessarily counting them but telling oneself, ‘ah, here I thought of something,’ and then, yes, really the fact of verbalizing what was felt.”* (P13)

One participant also highlighted the importance of knowing that she was not the only one experiencing a traumatic childbirth, noting that the existence of a study addressing childbirth trauma served to validate her feelings and normalize her experience.

*“I think it is the fact of talking about it, actually. The very fact of completing questionnaires where we talk about it, the effect of knowing that it happens—that I am not the only one. There are other people like me, and that is also why there is a study, in fact. So, I think knowing that being able to talk about it without… how can I say… really saying what we think, saying what we feel. Even now, the fact of having someone interested in listening to us… to listen to me about this has a quite positive impact, actually. In any case, I tell myself: ‘it is not trivial, what happened to me is still important!’”* (P09)

Completing the appointment, encompassing both a short childbirth narrative on the maternity ward and playing Tetris, was identified as a mechanism by six participants, as was returning to the maternity ward specifically by five participants. For some of these participants, the emotional intensity triggered by returning to the maternity ward was seen as an important part of the therapeutic process. Two participants described a broader process of becoming aware of the impact of their birth experience and allowing themselves to process previously blocked emotions.

*“I would even say it did me good to go back there, and to see that in a sense it is really not something bad. Everything goes well.”* (P17)

Among external factors, life changes such as separation from a partner or moving out, were mentioned by six participants, and the passage of time by five. Two participants also attributed part of their improvement to other therapeutic approaches that were not linked to their childbirth experience including antidepressant medication and osteopathy.

*“I am less emotionally affected by these flashbacks than at the beginning. But I don’t know, I have the feeling that it is the passing of time.”* (P03)

Importantly, for the seven participants who attributed changes to both intervention-related and external factors, these mechanisms were not perceived as mutually exclusive but rather as complementary contributors to their recovery.

## 4 Discussion

This qualitative study explored the subjective experiences of 17 women who received a single-session BI-VT targeting CB-IM combining narrating their childbirth in the maternity ward where they have given birth and a visuospatial task as part of a multicentric waitlist RCT (targeted *n* = 120). Three key findings emerged. First, recalling the traumatic childbirth memory in the maternity ward was experienced as emotionally intense, yet also as positive and meaningful. Second, participants reported a broad range of perceived changes following the intervention, most notably a reduction in CB-IM number and a deeper shift in their appraisal of the childbirth memory. Third, participants attributed these changes primarily to expressing and processing their childbirth experience.

### 4.1 Experience of the intervention

Recalling the traumatic childbirth memory (i.e., returning to the traumatic childbirth context and briefly narrating it) was predominantly experienced as emotionally intense, with most participants reporting negative emotions such as stress or sadness during this phase. This finding aligns with qualitative syntheses related to trauma-focused treatments’ experience, which highlight that revisiting traumatic experiences may elicit distress (Gjerstad et al., 2024; Lepisto et al., 2025). Importantly however, many participants simultaneously described the experience as moving and emotionally meaningful, suggesting a complex and ambivalent rather than purely distressing experience. This ambivalence mirrors findings from the broader trauma-focused treatment literature, where patients often retrospectively recognize emotional intensity as a necessary and productive part of the therapeutic process (Gjerstad et al., 2024).

The presence of the interviewer, who had also delivered the intervention, was spontaneously mentioned by several participants as having facilitated their experience on the maternity ward. This highlights the role of the therapeutic relationship as a meaningful therapeutic factor, consistent with research identifying non-specific factors such as feeling supported and safe as central to the experience of trauma-focused treatments (Gjerstad et al., 2024; Lepisto et al., 2025). The therapeutic relationship seems important in contributing to participants’ capacity to engage with emotionally challenging material.

Importantly, this capacity to engage with challenging aspects of the intervention was not only facilitated by the therapeutic relationship, but also by participants’ own active emotion regulation strategies. Allowing the expression of one’s emotions, rather than avoiding or suppressing them, was the most frequently reported individual coping strategy used when returning to the maternity ward, suggesting that participants drew on internal resources alongside relational support to navigate the emotional intensity of this phase.

It is worth noting that participants were not informed in advance that they would return to the maternity ward where they had given birth but only that they would briefly narrate their childbirth experience. However, this represents an intentional boundary condition of the intervention: informing participants in advance could lead them to anticipate and mentally reactivate the traumatic memory prior to the session, potentially prolonging the memory reactivation window beyond the timeframe required for reconsolidation-based mechanisms to operate effectively (Vaverkova et al., 2020). This may partly explain the challenging aspects of the traumatic childbirth memory recall reported by participants, including stress and fear, as the element of surprise may have amplified the intensity of returning to the trauma context. This lack of prior disclosure is somewhat unusual in the context of trauma-focused interventions, where patients are typically prepared for exposure to traumatic material. Nevertheless, a neutral room was available for each intervention should the therapist consider that the participant might not feel able to confront the maternity setting. Of note, Notably, none of the participants commented on not having been informed in advance that the intervention would involve a visit to the maternity ward during the interviews. However, as this aspect was not directly addressed in the interview guide, the absence of such comments should be interpreted with caution. The high acceptability of the intervention procedures reported in the pilot study (Deforges et al., 2022) nonetheless suggests that this aspect did not substantially interfere negatively with the participants’ intervention experience. Future studies are required to better understand the impact of returning to the maternity ward on the intervention’s perceived benefits, for instance by including a study arm in which the childbirth memory recall is conducted in a neutral location.

In contrast, playing Tetris was experienced quite differently, even though participants were also not informed about which visuospatial task they would engage in. The reaction to learning that they will play Tetris was characterized by an initial reaction of scepticism or surprise to engagement in the game. The predominantly positive experience of Tetris, despite initial doubts about its relevance, is noteworthy and suggests that this intervention component may serve a regulatory function following the emotional intensity of returning to the maternity and briefly narrating the childbirth, providing a cognitive shift that may facilitate emotional settling. Although this perceived regulatory effect does not reflect the theorized mechanisms of this intervention, this could be an unintended but beneficial side effect, which could contribute to the intervention efficacy.

### 4.2 Perceived changes following the intervention

Participants reported a broad range of perceived changes following the intervention, extending beyond CB-IM reduction. The most striking finding was the reduction in the number of CB-IM, reported by 15 of the 17 participants interviewed, with several describing the change as sudden and surprising. This finding is consistent with the preliminary quantitative evidence from the pilot study (Deforges et al., 2022) and supports the potential clinical significance of this intervention for CB-IM. Beyond frequency reduction, participants also reported qualitative changes in the characteristics of their CB-IM, including reductions in emotional intensity and nowness. This is congruent with studies reporting a reduction in IM-related distress and nowness following cognitive behavioural therapy (Hackmann et al., 2004), or BI-VT conducted in case studies (e.g., Lau-Zhu and Chan, 2025), although another study combining a traumatic memory recall with pharmacological agents to interfere with memory-updating processes did not find any change in IM-related characteristics (e.g., reliving, sensory modalities, emotions, and coherence) (Taïb et al., 2025). These changes are theoretically meaningful, as nowness and distress are specific IM characteristics that have been associated with PTSD severity (Evans et al., 2007; Michael et al., 2005), although evidence is mixed in relation to CB-IM (Fort et al., 2026), and further studies should explore the modifications of CB-IM characteristics following treatment.

Notably, participants described changes encompassing a broader transformation in their appraisal of the childbirth memory itself. Most participants reported greater acceptance of the childbirth memory, and additional participants reported an increased sense of control over the memory, and an ability to reframe their childbirth experience more positively. These changes suggest that the intervention may facilitate the processing of the traumatic childbirth experience, beyond its theorised effect on the memory trace (Meir Drexler and Wolf, 2018). Interestingly, negative appraisals of the traumatic event and a sense of loss of control over IM are recognized as key cognitive maintaining factors of PTSD (Ehlers and Clark, 2000). The observation that participants reported improvements on these dimensions suggests that the intervention may engage cognitive change mechanisms not explicitly targeted by its theoretical model: a finding that warrants further investigation in future quantitative studies examining appraisal-based outcomes.

The changes reported in CB-PTSS clusters beyond intrusions were more variable across participants. Participants described the most consistent improvements in negative mood, while irritability appeared unresponsive or even increased for a minority of participants. This differential pattern across symptom clusters is clinically relevant and warrants further investigation in future quantitative studies with adequate power to examine cluster-specific effects.

Additional changes were reported by some participants concerning their relationship with their child or partner, the possibility of considering a new pregnancy or improved sleep. Although these changes were reported in this study by a minority of participants, they reflect the negative impact that CB-PTSS may have on family relationships and daily functioning (e.g., Ayers et al., 2018; Van Sieleghem et al., 2022; Garthus-Niegel et al., 2018), supporting the need to develop accessible treatments. The results of this study are also congruent with the theorised role of CB-IM as a driver of broader symptom development and maintenance, and the hypothesis that targeting CB-IM could thus provoke broader benefits (McNally, 2012), although this warrants further investigation.

### 4.3 Perceived mechanisms of change

While the intervention was designed to reduce CB-IM through memory-updating mechanisms involving a visuospatial task (Deforges et al., 2022), none of the participants spontaneously identified this mechanism as central to their recovery. Instead, the most frequently reported perceived mechanism was talking about and writing down their childbirth experience. One explanation is that the memory-updating mechanism operates implicitly, below the level of conscious awareness, while participants attribute change to the more salient experiential components of the session, namely, the opportunity to narrate and process their childbirth experience in a supportive context. This interpretation is consistent with research suggesting that patients tend to attribute therapeutic change to interpersonal and narrative processes regardless of the specific theoretical model of the intervention (Lepisto et al., 2025).

The prominence of narrative and emotional processing in participants’ perceived mechanisms of change can be interpreted through the lens of the emotional processing theory (Foa and Kozak, 1986), which posits that therapeutic change in PTSS requires the activation and modification of the underlying fear memory structure. According to this framework, the act of narrating the traumatic childbirth experience may activate the fear structure in a way that allows corrective emotional information to be integrated, facilitating symptom reduction independently of the visuospatial interference mechanism. This interpretation is consistent with the broader literature on narrative-based approaches to trauma, including written exposure therapy, which achieves clinical benefits through repeated written narration of the traumatic event alone (DeJesus et al., 2024). The theoretical model introduced in the context of this intervention, namely memory-updating through visuospatial interference, may account well for the reduction in CB-IM frequency and qualitative characteristics reported by participants. However, it does not readily explain the broader changes in childbirth memory appraisal, the sense of acceptance, or the role of the therapeutic relationship and childbirth narration that participants identified as meaningful. These dimensions are better accounted for by complementary theoretical frameworks, including emotional processing theory (DeJesus et al., 2024) and the common factors model in psychotherapy (Wampold, 2015). A similar pattern has been observed in qualitative research on EMDR, another trauma-focused intervention used in the context of traumatic childbirth experience to alleviate CB-PTSS (e.g., Kopmeiners et al., 2023), in which patients emphasised the therapeutic relationship and the opportunity to process emotions and thoughts about the trauma as central to their recovery (Marich et al., 2020; Whitehouse, 2021). These finding highlight the need to examine in more detail not only the underlying mechanisms of the benefits perceived after this intervention in relation to the primary outcome, but also the role of other of non-specific therapeutic factors. To date, no study has evaluated each component of this intervention separately, which should be addressed in the future to improve our theoretical understanding and to support clinical refinement of this intervention (Elsey et al., 2018).

Returning to the maternity ward and briefly narrating the childbirth experience was also identified as a meaningful mechanism by several participants, who described the emotional intensity of this experience as therapeutic in itself, evoking a sense of confronting and working through unresolved material. This perception bears some resemblance to the rationale of exposure-based approaches, suggesting that the component of the intervention aiming at recalling the traumatic childbirth memory may function partly through exposure mechanisms in addition to, or instead of, memory-updating processes (Kida, 2020). However, the instructions given in this intervention differed from those typically given during exposure sessions, where patients are guided through repeated and prolonged recounting of the trauma narrative with the aim of fear reduction through habituation (Foa et al., 2019). In contrast, the memory reactivation in the present intervention was deliberately brief, serving as a cue to initiate memory-updating processes rather than to induce prolonged emotional processing. In addition, the therapeutic benefits reported by some participants when recording their CB-IM in a brief log (i.e., structured daily diary) are consistent with findings showing a reduction in IM following ecological momentary assessments of these symptoms over 2 weeks (Pollmann et al., 2024). As one participant noted, writing down CB-IM may provide an opportunity to *“re-analysing it afterwards, in a ‘cold’ way”*. Repeatedly documenting IM requires individuals to recall their sensory, emotional, and cognitive features, which may in turn facilitate the processing of traumatic memories, suggesting that these assessment may extend beyond simple symptom monitoring (Pollmann et al., 2024). However, the potential therapeutic benefits of reporting IM in daily diaries warrant further research (Zelkowitz et al., 2026).

Beyond the perceived mechanisms related to the childbirth memory, the combination of the childbirth narrative and playing Tetris was the second most frequently reported intervention-related mechanism of change. This suggests that, even if the theoretically intended mechanism was not identified, participants nonetheless attributed therapeutic value to Tetris as a component of the intervention. This perceived benefit may partly reflect a regulatory function of Tetris following the emotional intensity of the childbirth memory recall—an engaging (i.e., exciting and challenging) and predominantly positive experience (i.e., soothing and joyful) for participants that may have facilitated emotional settling. Finally, in general, participants attributed their changes to a combination of intervention-related and external factors, including the passage of time and concurrent life changes. This finding underscores the importance of contextualising intervention outcomes within participants’ broader life circumstances and highlights the limitations of attributing all perceived change to the intervention itself.

### 4.4 Strengths and limitations

The sampling strategy, based on self-reported changes in CB-IM, ensured the inclusion of participants reporting a range of experiences, including little or no improvement as well as symptom worsening. The interdependent coding process provided investigator triangulation, enhancing the credibility of the analysis. In addition, to our knowledge, this is the first qualitative study to explore participants’ experiences of this intervention, contributing novel insights into its subjective and potential therapeutic mechanisms. Finally, the study was embedded within a multicentre randomized controlled trial with a relatively large sample (targeted *n* = 120), strengthening the robustness and contextual relevance of the findings.

Several limitations should be acknowledged. The most significant one concerns the dual role of the interviewer, who had also delivered the intervention. First, participants may have been inclined to report more positive experiences or minimise negative ones out of a desire not to disappoint or contradict the person who had accompanied them through the intervention. This form of social desirability bias may be particularly salient when the interviewer holds a prior therapeutic role. Second, the interviewer’s familiarity with participants and her investment in the intervention may have unconsciously influenced the framing of questions or the depth of probing on certain topics, potentially amplifying positive accounts and limiting exploration of negative or ambivalent experiences. These risks are particularly relevant when interpreting findings related to perceived benefits, a domain especially susceptible to positive response bias. Several strategies were implemented to mitigate these risks: participants were explicitly reminded at the outset of each interview that there were no right or wrong answers; a second coder with no involvement in data collection independently coded all interviews, providing an external analytical perspective; and a reflexive journal was maintained throughout to document and critically examine the interviewer’s potential influence on the data. Nonetheless, the possibility that social desirability bias contributed to the predominantly positive findings of this study cannot be entirely ruled out, and results should be interpreted accordingly, alongside the quantitative evaluation supporting the intervention’s effects (Deforges et al., 2022). The uneven distribution of the sample, with 15 of 17 participants reporting symptom improvement, further limits the representativeness of findings for women who did not benefit from the intervention. Increasing the overall sample size in future studies would allow investigation of more potentially heterogeneous responses to this intervention. Furthermore, the single question used to assess self-reported change in CB-IM number for the maximum variation sampling strategy may lack sensitivity in identifying participants with little or no change in their symptoms. Future studies should consider alternative strategies, such as inviting the entire sample to participate in interviews, or adjusting the cut-off used in this question to maximise variation and support data saturation. Another limitation is that interviews were only conducted with participants who received the intervention. Thus, the interpretations of the reported intervention’s perceived benefits should be carefully considered in relation to other potential factors such as the passage of time and non-specific therapeutic factors (e.g., the therapeutic relationship). Future studies should also consider strategies to minimise retrospective memory bias arising from the time elapsed between the intervention and the interviews (i.e., up to 77 days in the present study). To this end, interview questions could be administered at different timepoints corresponding to each research question (i.e., experience of the intervention, perceived effects, perceived mechanisms of change), or open-ended questionnaires could be completed by participants in the days immediately following the intervention. Additionally, member checking, whereby participants review and validate the researcher’s interpretations of their accounts, could be implemented to enhance the credibility of the findings and reduce the risk of misrepresentation.

### 4.5 Clinical implications

The emotional intensity of the brief childbirth narrative on the maternity ward points toward the importance of the presence of a clinician with an adequate training to support participants during the intervention.

The finding that women most frequently identified briefly recounting their childbirth experience and recording their CB-IM in a daily log as mechanisms of change suggests that the narrative component of the intervention may be particularly meaningful to them. Although the brief childbirth recall was originally intended to trigger memory-updating processes, participants’ accounts indicate that it may also provide a valuable opportunity to process and express their experience. This highlights the importance of offering space for women who have experienced traumatic childbirth to express their experience.

The broad range of perceived changes reported, extending to general wellbeing, suggests that the intervention may have a wider impact than currently captured by standardised CB-PTSS outcome measures. Future studies should consider incorporating patient-reported outcomes measuring different aspects of quality of life that reflect these broader dimensions of recovery.

## 5 Conclusion

This study provides the first qualitative account of women’s experiences of a single-session BI-VT combining traumatic childbirth memory recall and a visuospatial task (Tetris) to target CB-IM. Findings suggest that the intervention is experienced as emotionally intense yet meaningful and is associated with a broad range of perceived changes extending beyond CB-IM reduction to encompass broader shifts in women’s appraisal of their childbirth experience. Participants primarily attributed the therapeutic benefits to interpersonal and narrative processes, highlighting the importance of non-specific therapeutic factors alongside the theorised mechanisms of this intervention.

## Data Availability

The data that support the findings of this study are not publicly available due to the sensitive nature of the qualitative data and the need to protect participant confidentiality but may be available from the corresponding author upon reasonable request and with appropriate ethical approvals. Requests to access the datasets should be directed to antje.horsch@chuv.ch.
